# Sub-wavelength scale randomly frozen microbubble during short-pulsed-ultrasound-driven microbubble cluster dynamics in microfluidic channel

**DOI:** 10.1016/j.ultsonch.2026.107912

**Published:** 2026-06-02

**Authors:** Yi Xu, Siyu Luo, Yujie Wang, Liying Wang, Yuzhe Fan, Fenfang Li

**Affiliations:** a School of Basic Medical Sciences, Beihua University, Jilin City, China; b Institute of Molecular Physiology, Shenzhen Bay Laboratory, Shenzhen, China; c School of Biology and Biological Engineering, South China University of Technology, Guangzhou 510006, China; d Institute of Physics, Otto-von-Guericke University Magdeburg, Germany

**Keywords:** Acoustic cavitation, Vessel-mimicking microchannels, Flow velocity, Short pulse ultrasound, Duty cycle

## Abstract

Microbubble cloud dynamics under short-pulse ultrasound in flowing environments remain insufficiently understood. Here, we investigate microbubble cloud behaviour in a vessel-mimicking microfluidic channel under short-pulse ultrasound (1.125 MHz) and controlled laminar flow (37.5–150 µL/min). High-speed visualization reveals two distinct regimes: an actively interacting regime characterized by clustering and coalescence, and a ‘frozen’ regime in which microbubbles exhibit minimal displacement despite continued ultrasound excitation. Both regimes lead to the formation of spatially frozen, yet oscillating, microbubbles at the subwavelength scale. We observe clustered frozen microbubbles in the frozen region and isolated relative larger microbubble in the actively interacting region. A theoretical model including hydrodynamic drag with wall correction captures the transition between actively moving and frozen states. The results indicate that microbubble cloud dynamics under short-pulse excitation is determined by a dynamic competition between acoustic radiation forces and near-wall hydrodynamic drag, with flow rate and pulse duration acting as coupled control parameters.

## 1 Introduction

Microbubble-assisted non-thermal focused ultrasound therapy has attracted increasing attention in recent years because of its unique advantages in brain drug delivery, tissue ablation, and immune modulation [1]. In this approach, coated microbubbles, also known as ultrasound contrast agents (UCAs) [2], [3], are injected into the body as exogenous cavitation nuclei [4]. Under ultrasound excitation, these microbubbles can undergo stable oscillations accompanied by cyclic jet formation [5], or they can experience inertial cavitation, in which excessive expansion during rarefaction is followed by violent collapse. Non-inertial cavitation is generally preferred for producing subtle and transient biological effects, whereas inertial cavitation can be exploited to induce localized tissue damage [6], [7], [8], [9].

Although single-microbubble dynamics and their interactions with surrounding tissues or phantoms have been extensively investigated, exogenous microbubbles may exist in vivo as bubble clouds rather than isolated entities, e.g., in large-diameter blood vessels. Within such clouds, complex bubble–bubble interactions give rise to large secondary acoustic emissions [10], [11], [12], [13], driving microbubble migration [14], [15], [16], clustering [17], [18], and coalescence [19]. In particular, acoustic radiation forces generated by the external ultrasound field or by oscillating neighboring bubbles can induce pronounced clustering of microbubbles [20] and enhance UCA attachment to target surfaces [21]. These processes suggest that ultrasound can strongly influence the spatiotemporal structure of bubble clouds and thereby modulate therapeutic outcomes.

In most preclinical and clinical studies, long-pulse (LP) ultrasound sequences are employed, as robust therapeutic effects can be achieved by prolonging acoustic exposure to open the endothelial barrier [22]. However, these approaches are also associated with adverse bioeffects, including erythrocyte extravasation [23], edema [24], and neuroinflammation [25]. In contrast, emerging short-pulse (SP) ultrasound protocols, characterized by brief and repetitive bursts, deliver substantially less total energy and have shown promise for achieving more uniform and reversible barrier opening with minimal tissue damage [26], [27], [28], [29]. Under short-pulse operation, appropriate adjustment of the duty cycle can balance microbubble replenishment with acoustic energy input, which may be achieved through reducing fragmentation, clustering and coalescence [30], [31]. However, a critical gap remains in our understanding of the intricate interplay of cavitation dynamics under varying physiologically relevant flow conditions, both within a single short pulse and across successive short pulses. Only a few studies have employed high-speed microscopy to resolve microbubble dynamics under short pulse sequences [31]. Yet there is a lack of spatiotemporal resolution to capture both individual bubble and bubble cloud behavior under short-pulse ultrasound excitation. In addition, in physiological environments, hemodynamic flow conditions vary widely and directly modulate bubble trajectories, spatial distribution, and residence time [32]. Clinically, hemodynamic characteristics differ substantially across pathological tissues [33]. For example, tumor vasculature exhibits reduced flow velocities (5–15 mm/s) compared with normal microvascular flow due to structural abnormalities and elevated vascular resistance [33], [34], while ischemic regions exhibit even slower flow [35]. Such hemodynamic heterogeneity strongly influences bubble–flow and bubble–cell interactions [36], [37], [38], [39]. Nevertheless, the explicit relationship between ultrasound, particularly short-pulse sequences, and microbubble dynamics under varying flow conditions remains poorly understood [32], [40], [41], [42].

In this work, we employ an integrated microfluidic–acoustic platform to investigate the coupled effects of flow rate and ultrasound pulse duration on microbubble dynamics under short-pulse excitation. Microfluidic systems provide a well-controlled experimental framework for reconstructing vascular-scale environments and precisely regulating flow velocity and shear stress [43], [44], [45], [46], while enabling high-resolution visualization of bubble dynamics [47], [48], [49], [50]. Using high-speed imaging and quantitative analysis, we systematically characterize individual bubble and bubble cloud behavior across a range of flow rates and duty cycles. Our results reveal competition between acoustic radiation forces and hydrodynamic drag during microbubble clustering and coalescence and demonstrate that flow velocity can determine the microbubble cloud dynamics near the wall.

## 2 Materials and methods

### 2.1 Vessel-mimicking microchannel fabrication

**Fig. 1:**
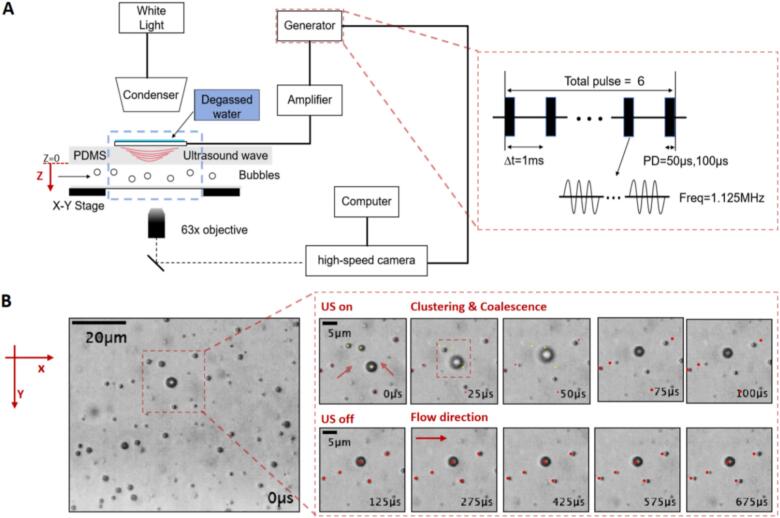
**Experimental setup and bubble dynamics recording**. (A) Experimental setup and schematic of the short-pulse ultrasound sequence used in the experiments. (B) High-speed snapshots of bubble dynamics under ultrasound excitation and fluid flow (flow rate: 37.5 µL/min; pulse length: 50 µs). Yellow dots indicate the initial positions of bubbles that subsequently coalesced, whereas red dots indicate the initial positions of bubbles that did not coalesce (top row on the right). Bubbles cluster and coalesce during ultrasound excitation and move downstream with the flow once the ultrasound is turned off.

Each microfluidic channel used in this study is 200 µm in width, 100 µm in height, and 17000 µm in length. The PDMS wall on top of the channels is made 3 mm thick to align the ultrasound beam focus at the channel in the Z direction (Fig. 1A). Marker patterns were integrated in the design of microchannels, allowing precise X-Y positioning and focus of the ring ultrasound transducer to the channels (Fig. S2 in the Appendix). The microchannels mimicking vascular structures were designed using SU-8 master mold produced through standard soft lithography techniques. A 10:1 mixture of polydimethylsiloxane (PDMS, Sylgard 184 Silicone Elastomer Kit, Dow Corning) was poured onto the mold, cured at 60 °C for 4 h, and subsequently bonded to #1 cover glass slides (25 × 50 mm^2^) immediately after a 50 s plasma treatment (Diener, Zepto one).

### 2.2 Preparation and characterization of microbubbles

Microbubbles composed of a lipid shell and filled with perfluoropropane gas were generated according to previously described methods [51]. The lipid formulation consisted of 1,2-distearoyl-*sn*-glycero-3-phosphocholine (DSPC) and N-(carbonyl-methoxypolyethylene glycol-2000)-1,2-distearoyl-*sn*-glycero-3-phosphoethanolamine (DSPE-PEG2000) (Lipoid, Ludwigshafen, Germany) in a 9:1 M ratio. After the synthesis process, the microbubbles were diluted with Isoton II solution, and their concentration and size distribution were measured using a Coulter Counter Multisizer IV (Beckman Coulter Inc., USA). Their morphology was then examined under a microscope (Zeiss, Axio Observer 7). The size distribution and stability of the home-made microbubbles is given in Fig. S1 in Appendix A and is like previous studies [52].

### 2.3 Setup for recording bubble dynamics in microchannels under ultrasound exposure and varying flow conditions

For the bubble dynamics experiments, previously prepared polydisperse microbubbles were diluted 1:20 (v/v) in 1 × Dulbecco’s phosphate-buffered saline (DPBS) and introduced into the microchannels at flow rates of 37.5, 75 and 150 µL/min using a syringe pump (KDS, R462) mounted on an inverted microscope (Zeiss, Axio Observer 7), as shown in Fig. 1A. A PZT-4 ring-shaped ultrasound transducer (center frequency 1.125 MHz; inner diameter 7 mm, outer diameter 10 mm, height 1.2 mm) was designed and operated in thickness mode [53]. Its open-center design was chosen to permit sufficient transmission of microscope condenser light for high-speed bright-field imaging of bubble dynamics. Sonication was performed using this custom-built ring-shaped ultrasound transducer operating at 1.125 MHz (corresponding to a wavelength of λ ∼ 1.33 mm in the microchannel medium) and driven by a 50-dB power amplifier (2100 L, Electronics & Innovation, USA). The transducer was aligned on top of the PDMS chip with designed markers using high-vacuum grease (Dow Corning) as coupling gel. The driving ultrasound signals were generated by one function generator (DG972, RIGOL, China). The measured acoustic field is given in Appendix B. The acoustic pressure output through the PDMS layer was calibrated with a hydrophone (HNR-0500, Onda Corporation, USA). A short-pulse ultrasound sequence was applied (Fig. 1A *right*) with an effective peak pressure estimated to be of 0.15 MPa [5], [54], a pulse repetition frequency of 1 kHz, and pulse lengths ranging from 50 to 100 µs. Bubble motion was imaged under a 63 × objective (LD PN 63×/0.75 Corr) using a high-speed camera (Photron, Nova S12) that was synchronized to each ultrasound pulses. The camera operated at either 40,000 frames per second or 750,000 frames per second with an exposure time of 0.66 µs or 0.33 µs, respectively.

### 2.4 Qualification and statistical analysis

High-speed videos of bubble dynamics were imported into MATLAB (The MathWorks, Natick, MA, USA; academic license) for quantitative analysis of microbubble number and motion. The recorded images were first preprocessed using the ‘imflatfield’ function to correct for nonuniform background illumination, followed by median filtering function ‘medfilt2′ to suppress high-frequency noise. Subsequently, images were binarized using the adaptive thresholding function ‘adaptthresh’. Microbubble number and centroid positions were extracted from the binary images using the ‘regionprops’ function.

For microbubble tracking, a nearest-neighbor approach was employed. The position of each bubble in the current frame was matched to the closest bubble in the subsequent frame based on the minimum Euclidean distance criterion. A match was accepted when the minimum-distance condition was satisfied [55].

Significant differences were determined by un-paired Student *t*-test for comparison between two groups.

## 3 Results

### 3.1 The influence of ultrasound and laminar flow rate on bubble dynamics nearby the PDMS surface

In this study, we focus on microbubble cluster dynamics near the upper PDMS wall (Fig. 1A). As shown in Fig. 1B (left), the freshly synthesized microbubbles exhibit a polydisperse size distribution, oscillate under ultrasound excitation, and move downstream with the laminar flow parallel to the wall. When ultrasound is applied, bubbles oscillate and may exhibit a tendency to migrate toward one another, leading to clustering. Microbubbles located in the central region of the image (within the small red dashed box and original position marked in yellow (0–50 µs)) coalesce, while surrounding bubbles migrate toward this region (original position labeled by red dots, Fig. 1B, right top). Once the ultrasound is turned off, the microbubbles resume downstream motion with the background flow at velocities much lower than those observed during ultrasound excitation (Fig. 1B, right bottom).

**Fig. 2:**
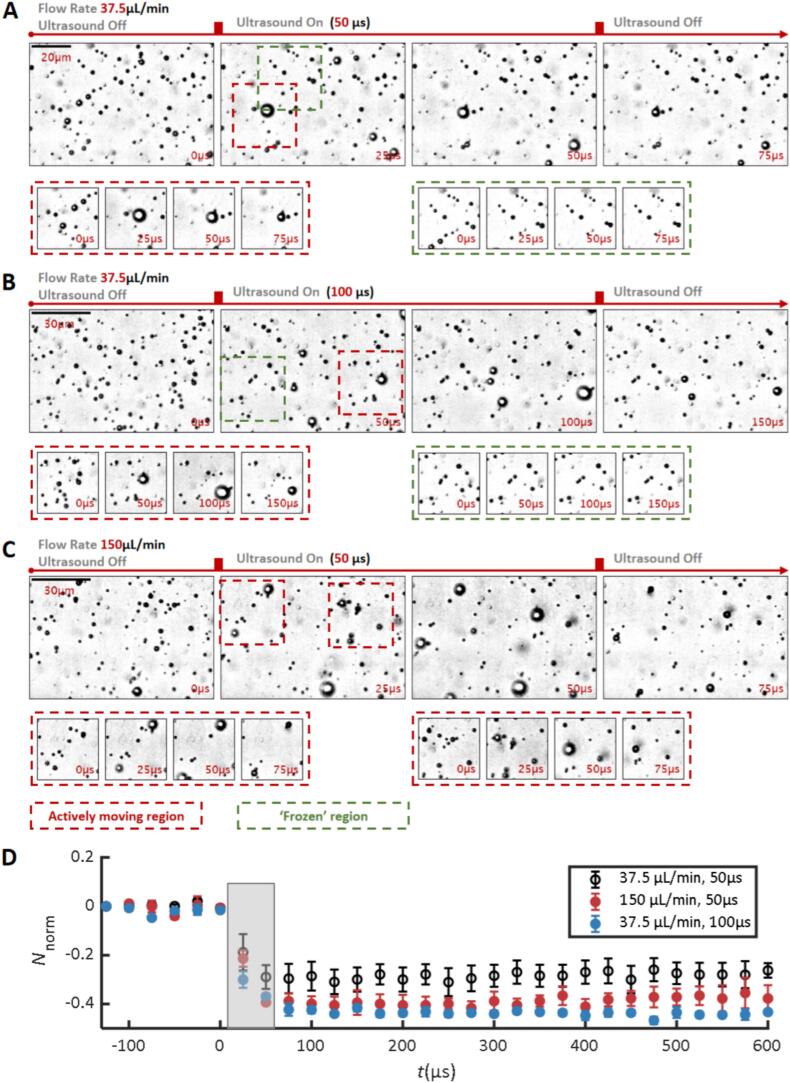
**Analysis of bubble motion and bubble dynamics under a single shot-pulse ultrasound sequence.** Selected images of bubble motion and dynamics under ultrasound excitation: (A) flow rate 37.5 µL/min, pulse length 50 µs; (B) flow rate 37.5 µL/min, pulse length 100 µs; (C) flow rate 150 µL/min, pulse length 50 µs. The dashed red and dashed green boxes mark the actively moving region and the 'frozen'” region of microbubbles during ultrasound excitation, respectively. (D) Microbubble number as a function of time under different conditions. The shaded region indicates the application of the 1st ultrasound pulse. Error bars indicate the standard deviation obtained from independent realizations. Each data represents Mean ± SEM. Three individual videos are analyzed in each of the three conditions.

Fig. 2 demonstrates that both flow rate and ultrasound pulse length strongly influence bubble dynamics in sub-wavelength scale. In Fig. 2A (flow rate 37.5 µL/min; pulse length 50 µs), bubbles exhibit pronounced motion during ultrasound exposure; however, a 'frozen' region is observed in which microbubbles display minimal displacement. A similar spatial heterogeneity is observed for a longer pulse length (Fig. 2B; flow rate 37.5 µL/min; pulse length 100 µs). The longer pulse duration allows more time for in-phase oscillating microbubbles to attract each other, leading to an increased number of coalescence events in the actively moving region (Fig. 2B, red dashed box). Such frozen regions occur randomly in sub-wavelength scale (smaller than 0.1λ) during experiments in each realization. These observations and analysis strongly suggest that microbubbles are not trapped by the acoustic field. In contrast, at a higher flow rate (Fig. 2C; 150 µL/min; pulse length 50 µs), most bubbles respond actively to ultrasound excitation. Under this condition, smaller microbubbles tend to coalesce locally in multiple regions, resulting in the formation of several larger bubbles distributed across the channel.

To quantify these observations, we analyzed the normalized bubble number, $N_{\mathrm{norm}}\left(t\right)=\left(N\left(t\right)-N_{0}\right)/N_{0}$, where $N_{0}$ is the average microbubble number during the 200 μs baseline recording before ultrasound, and N(t) is the bubble count in each recorded frame. As shown in Fig. 2D, both increasing pulse length and increasing flow rate result in a larger drop of the normalized bubble number after 1 burst of ultrasound sequence, and a single pulse excitation already produces an approximately 10% change in $N_{\mathrm{norm}}\left(t\right)$.

**Fig. 3:**
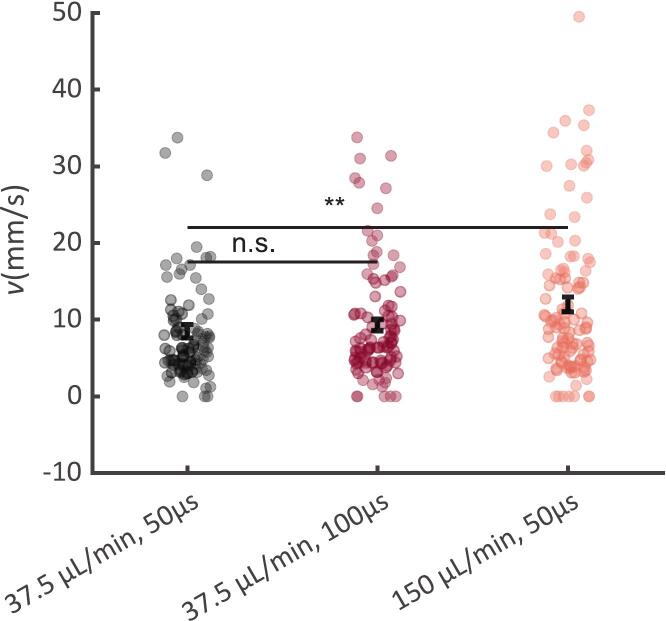
**Statistical analysis of microbubble velocity under ultrasound excitation.** The average microbubble velocity v during the first 50 µs of ultrasound exposure was quantified. The analysis was restricted to microbubbles that did not undergo coalescence during excitation. Three individual videos are analyzed in each condition. Unpaired student *t* test is used for statistical analysis on the entire dataset. The number of bubbles analyzed in each group is 98, 106 and 98 from left to right.

We further quantified the average velocity of microbubbles (over the first 50 µs during ultrasound excitation) that did not undergo coalescence during the ultrasound excitation (Fig. 3). The results indicate that the mean microbubble velocity at the higher flow rate (150 µL/min) is statistically significantly greater (p < 0.01) than that at the lower flow rate (37.5 µL/min). The measurements quantitatively confirm that microbubbles exhibit stronger motion under ultrasound excitation at higher flow rates. Moreover, although a longer pulse duration leads to a decrease in the normalized bubble number, it has little influence on the average velocity of non-coalesced microbubbles. This indicates that, under low-flow-rate conditions, the pulse duration has minimal influence on the 'frozen' zone. One must notice that the direction of bubble motion during ultrasound exposure is not aligned with the background flow and appears random. Moreover, the bubble velocities under ultrasound are substantially greater than the laminar flow velocity. Therefore, the increased flow rate does not directly drive the enhanced bubble motion observed during ultrasound excitation.

### 3.2 Microbubble dynamics after multiple short-pulse ultrasound

**Fig. 4:**
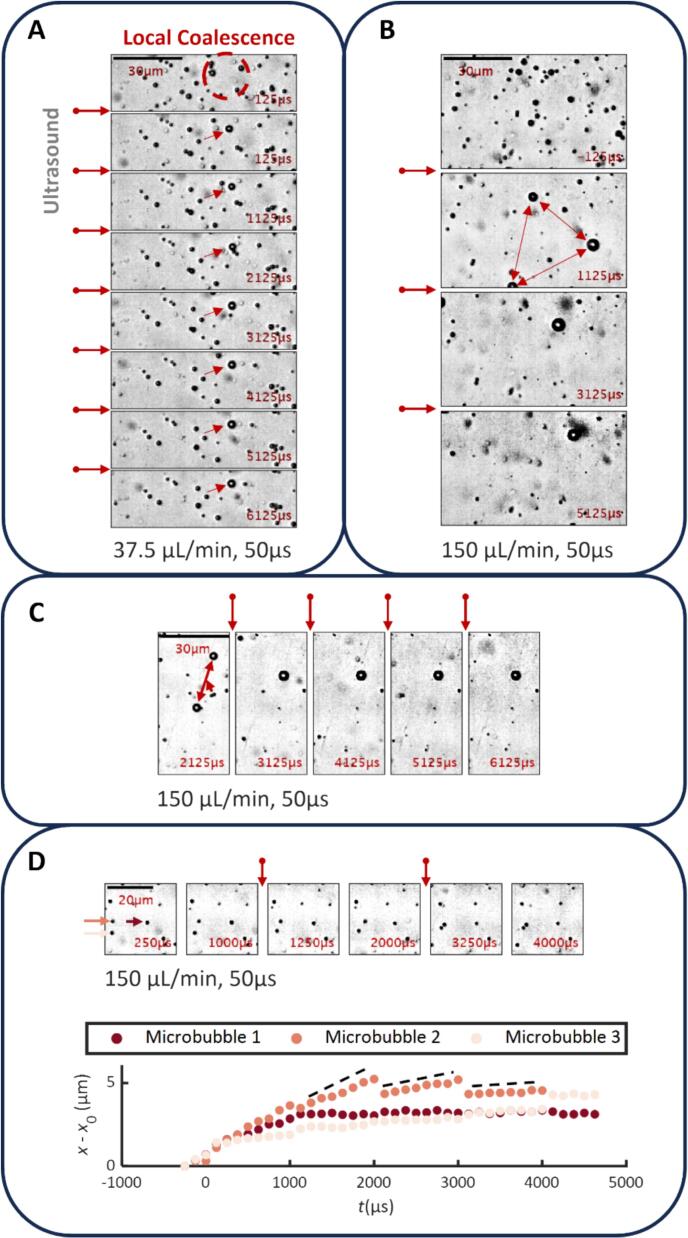
**Typical scenarios of microbubble cloud dynamics under multiple short-pulse ultrasound sequences.** Selected high-speed snapshots of bubble dynamics under (A) low flow rate (37.5 μL/min) and (B) high flow rate (150 μL/min). The pulse length is 50 µs for all cases shown. Under low flow rate, bubbles within a localized region (red dashed outline) coalesce and form a larger microbubble (radius ≈ 1.7 µm, pointed to by the red arrow). This large microbubble subsequently attracts surrounding microbubbles, which migrate slowly toward it. Under high flow rate, large microbubbles form in multiple regions after the first ultrasound burst and interact with each other over longer distances. After several short-pulse sequences, these bubbles coalesce into a big microbubble (radius ≈ 3.2 µm), effectively clearing microbubbles from the surrounding area. (C) Coalescence of two large microbubbles and two tiny bubbles under flow rate of 150 μl/min and ultrasound pulse length of 50 μs. After coalescence, the larger microbubble remains stationary at its position and ceases downstream motion. (D) The microbubble dynamics in ‘frozen’ region. The displacements of three representative microbubbles (indicated by colored arrows in the top row at 250 μs) are quantified in the bottom figure (x-x_0_ vs. t) using corresponding colors. Bubble 1 within the ‘frozen’ region exhibit minimum movement under ultrasound excitation and progressively decreasing downstream velocities. For microbubble 2, the velocity drops markedly between 1000 µs and 3000 µs (dashed line) and it coalesces with microbubble 3 at approximately 4000 µs. After 4000 µs, both bubbles remain stationary.

The typical evolution of bubble dynamics is shown in Fig. 4, where short-pulse ultrasound bursts are emitted every 1 ms. Under low flow rate (Fig. 4A, 37.5 µL/min), only bubbles within a relatively small region (e.g., marked by a red dashed circle with a radius of 10 µm) coalesce after the first ultrasound burst, forming a larger microbubble (radius ≈ 1.7 µm). Subsequently, this larger microbubble gradually attracts surrounding smaller bubbles toward itself after each ultrasound burst. In contrast, under high flow rate (Fig. 4B, 150 µL/min), microbubbles coalesce in multiple regions, leading to the formation of several large microbubbles (average radius ≈ 2.2 µm, at 1125 µs). These larger bubbles interact with each other over longer distances during ultrasound excitation, exhibit pronounced motion, and progressively clear microbubbles from the surrounding area. Eventually, they merge into a big microbubble (radius ≈ 3.2 µm).

We also observe that microbubbles can become ‘frozen’ after several ultrasound bursts, both at low flow rate (Fig. 4A, 37.5 µL/min) and at high flow rate (Fig. 4C, 150 µL/min). Both Fig. 4C and Fig. 4A show that, once a large microbubble forms, it can remain stationary near its formation point and ceases downstream motion. Such an effect is not unique to large microbubbles but also exist in the ‘frozen’ zone (Fig. 4D). The trajectories of three representative microbubbles are shown in Fig. 4D bottom, with corresponding-colored arrows labeled in Fig. 4D. Microbubbles within the ‘frozen’ region exhibit minimum movement under ultrasound excitation and progressively decreasing downstream velocities. Especially for microbubble 2, the velocity drops markedly between 1000 µs and 3000 µs (dashed line) and it coalesces with microbubble 3 at approximately 4000 µs. After 4000 µs, both bubbles remain stationary.

**Fig. 5:**
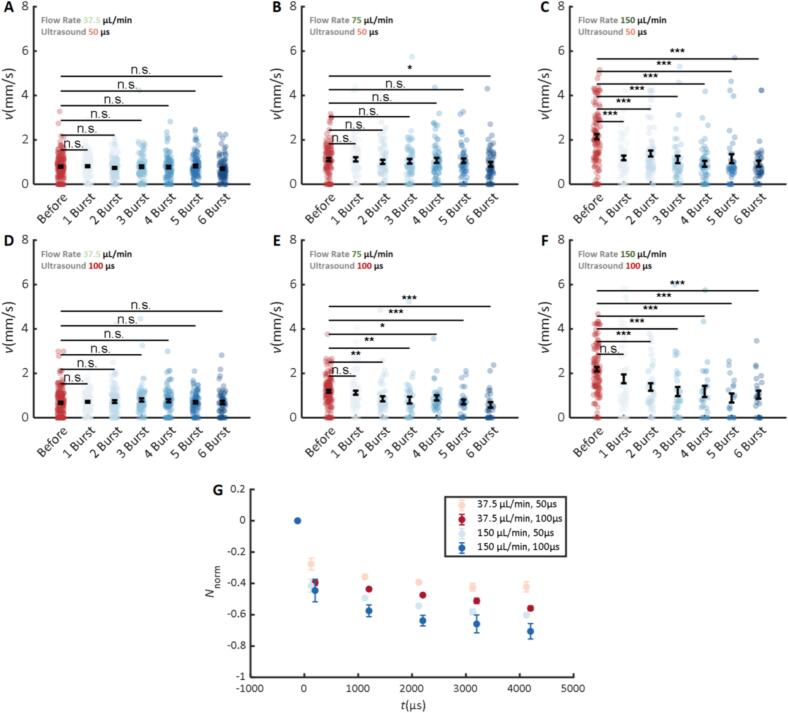
**Statistical analysis of microbubble velocity under different conditions.** (A) flow rate 37.5 µL/min, pulse length 50 µs; (B) flow rate 75 µL/min, pulse length 50 µs; (C) flow rate 150 µL/min, pulse length 50 µs; (D) flow rate 37.5 µL/min, pulse length 100 µs; (E) flow rate 75 µL/min, pulse length 100 µs; (F) flow rate 150 µL/min, pulse length 100 µs. (G) Microbubble number as a function of time under different conditions. Error bars indicate the standard deviation obtained from independent realizations. Error bars represent Mean ± SEM. Three individual videos are analyzed in each condition.

Experimental observations indicate that the low flow rate promotes the development of clustered frozen microbubbles, and high flow rate promotes the development of isolated relatively large microbubbles. This trend is further quantified by measuring the average bubble velocity after each ultrasound burst (Fig. 5). At high flow rate (Fig. 5C, F; 150 µL/min), ultrasound significantly reduces bubble velocity from the 2nd burst (p < 0.001), indicating that microbubbles get ‘frozen’ by short-pulse ultrasound exposure. In contrast, at low flow rate (Fig. 5A, D; 37.5 µL/min), ultrasound has minimal influence on bubble motion, implying that the ‘frozen’ microbubbles are developed mostly by the low flow rate itself. At intermediate flow rate (Fig. 5B, E; 75 µL/min), a moderate reduction in velocity is observed, particularly under longer pulse duration (100 µs), indicating a combined effect of flow rate and ultrasound exposure. Fig. 5G further shows that increasing both pulse length and flow rate leads to a greater reduction in the normalized bubble number, $N_{\mathrm{norm}}\left(t\right)$*.*

### 3.3 The dynamics of frozen microbubbles

We first analyze the oscillation of the frozen microbubbles. We consider the time-dependent bubble radial oscillation under traveling-wave excitation, which can be described by a Rayleigh–Plesset-type equation [13], [56], [57]

(1) $$\rho \left(\ddot{R}R+\frac{3}{2}{\dot{R}}^{2}\right)=\left(1+\frac{R}{c}\frac{d}{\mathrm{dt}}\right)P_{g}-P_{0}-P_{A}-\frac{2\sigma \left(R\right)}{R}-\frac{4\kappa_{S}\dot{R}}{R^{2}}-\frac{4\mu \dot{R}}{R}$$

Where $P_{g}=\left(P_{0}+\frac{2\sigma (R)}{R_{0}}\right){\left(\frac{R_{0}}{R}\right)}^{3\kappa }$ is the gas pressure inside the bubble, ρ=998.7 kg/m^3^ is the liquid density, c=1482 m/s the speed of sound in the liquid, κ = 1.4 the polytropic exponent of the gas inside the bubble, $P_{0}=101325$ Pa the local hydrodynamic pressure within the channel and $P_{A}\left(t\right)=-p_{A}sin\left(2\pi ft\right)$, where f=1.125 MHz and with $p_{A}$ the acoustic pressure amplitude, µ = 0.001 Pa·s is the dynamic viscosity. $R_{0}$ is the initial bubble radius, R is the time-dependent radius of the bubble and the overdots denote its time derivatives. Further, $\sigma \left(R\right)$ represents the interfacial tension and is given as:

(2) $$\sigma \left(R\right)=\left\{\begin{array}{cc} 0 & \mathrm{forR}\leq R_{\mathrm{buckling}},\\ \sigma \left(R_{0}\right)+\chi \left(\frac{R^{2}}{R_{0}^{2}}-1\right) & \mathrm{for}R_{\mathrm{buckling}}\leq R\leq R_{\mathrm{rupture},}\\ \sigma_{\mathrm{water}} & \mathrm{forR}\geq R_{\mathrm{rupture}}. \end{array}\right)$$

**Fig. 6:**
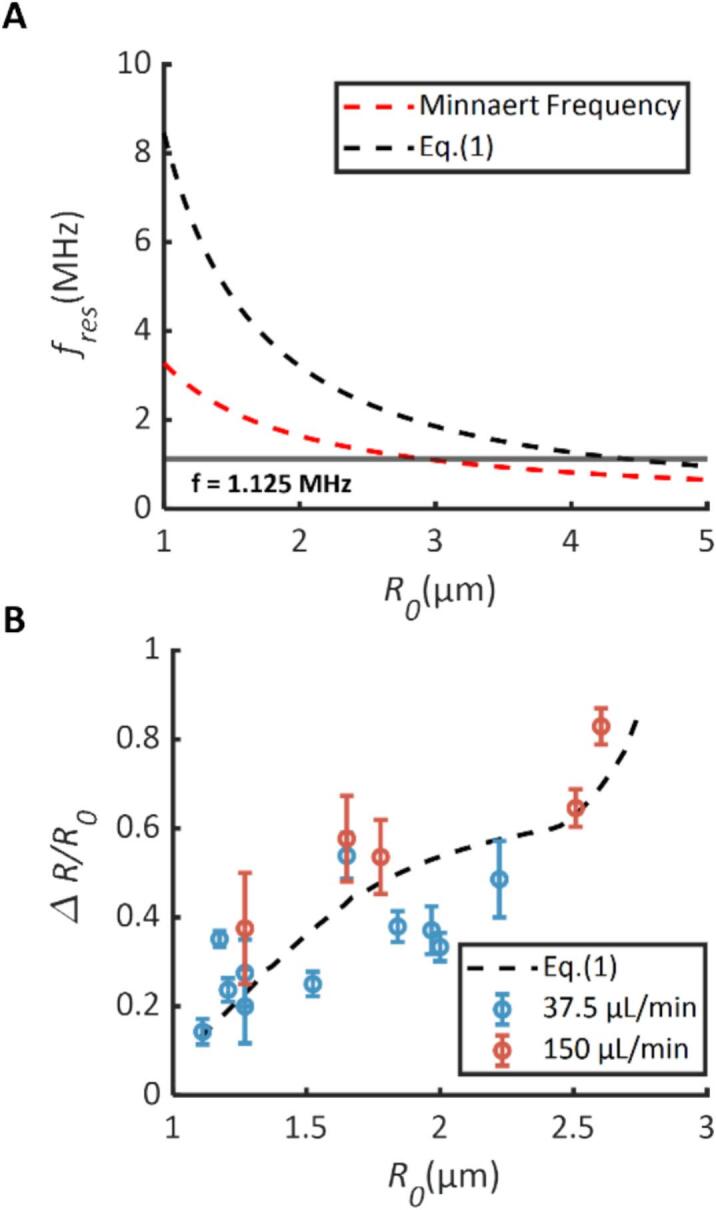
**The resonant frequency and the volume oscillation of microbubbles.** (A) The dependence of bubble natural frequency $f_{\mathrm{res}}$ on the equilibrium bubble radius $R_{0}$. (B) The dependence of normalized radial expansion of microbubbles $\frac{\Delta R}{R_{0}}$ on the equilibrium bubble radius $R_{0}$. Error bars represent Mean ± std. The data points are obtained from experiments while the dashed line is acquired by numerical modeling using Eq. (1).

The surface tension $\sigma \left(R\right)$ is defined as zero for radii below the bucking threshold, matching the surface tension of a clean gas–water interface, is defined as $\sigma_{\mathrm{water}}=0.072$ N/m beyond the rupture radius and varies linearly with the relative area deformation in the intermediate regime ($R_{\mathrm{buckling}}\leq R\leq R_{\mathrm{rupture}}$) [57], [58]. In the simulation, $\sigma (R_{0})=0$, $\kappa_{S}=10\times 10^{-9}$ kg/s and χ=0.6 N/m is adopted [59], [60], [61]. By linearizing Eq. (1), the nature frequency of microbubbles can be given as $f_{\mathrm{res}}=\frac{1}{2\pi }\sqrt{\frac{1}{\rho {R_{0}}^{2}}\left(3\kappa P_{0}+\frac{4\chi }{R_{0}}\right)}$ [56], [62], and $f_{\mathrm{res}}$ reduces to Minnaert frequency when χ=0. Fig. 6A shows the dependence of nature frequency on equilibrium microbubble radius, indicating that $f_{\mathrm{res}}>f$ for the studied bubble, where the observed maximum microbubble radius are 3.5 µm (after coalescence). Fig. 6B shows the dependence of the normalized radial expansion of isolated frozen microbubbles under different flow rates, where $\Delta R=R_{\max}-R_{0}$. The ultrasound pressure is experimentally measured using a hydrophone (Appendix B), and in simulation is adjusted to fit the experimental measurement to account for the influence of the complex acoustic environment on microbubble dynamics. The results show that the bubble oscillation is enhanced with the increasing of microbubble radius and confirm that the flow rate does not significantly contribute to the bubble oscillation.

### 3.4 The microbubble motion parallel to the wall

To understand the development of frozen microbubbles, the forces acting on a microbubble during a short-pulse ultrasound burst is given as [63]:

(3) $$0=F_{R}+F_{A}+F_{D}+F_{\mathrm{his}}$$

where, $F_{R}=-V\nabla P_{R}$ denotes the secondary Bjerknes force arising from the acoustic emissions of neighboring microbubbles, where V is the time-dependent bubble volume, and $P_{R}$ is the pressure wave emitted by the neighbor microbubbles. Since $f_{\mathrm{res}}>f$ is kept in the current study and microbubbles are under mild oscillation, the radiation force $F_{R}$ attracts in-phase oscillating microbubble moving towards each other [64], [65], and is counteracted by the hydrodynamic drag force near the wall, given by $F_{D}=\frac{16}{5}\pi \mu UR_{0}\ln\left(h/R_{0}\right)$ [66], where U is the transverse velocity of the microbubble parallel to PDMS wall, $h=h_{0}-R_{0}$, where $h_{0}$ is the distance between microbubble center to the wall, and an added mass force $F_{A}=\frac{1}{2}\rho \frac{d}{\mathrm{dt}}\left(\frac{4\pi }{3}\dot{R}U\right)$ arises from the acceleration of the surrounding fluid [67]. The surface of coated microbubble can be approximated as a rigid sphere [63]. When a smooth particle approaches a wall, lubrication theory predicts a hydrodynamic singularity that prevents the particle from translating at finite forces [68], [69]. It indicates that as the normalized gap $h/R_{0}$ decreases, the hydrodynamic drag increases, e.g., when *h* approaches 0, the drag would become*∞*, effectively suppressing bubble motion (with considerably declined U) parallel to the PDMS wall. The history force $F_{\mathrm{his}}$ arises due to the unsteady diffusion of vorticity from the bubble surface [70], given by $F_{\mathrm{his}}=-6\pi \mu \int_{-\infty }^{t}\frac{1}{\sqrt{\pi \nu \int_{\tau }^{t}{R(s)}^{-2}ds}}\frac{d(R\dot{x})}{d\tau }d\tau$, where ν is the fluid kinematic viscosity.

**Fig. 7:**
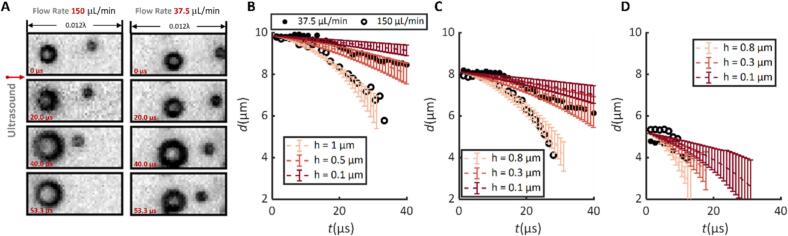
**Experimental and simulation results of the bubble motion parallel to the wall.** (A) Selected high-speed experimental images showing the motion of an unequal-sized microbubble pair under different flow rate. During the ultrasound excitation, the small microbubble is attracted by the large one. (B-D) The comparison between experimental and simulation results on bubble–bubble distance d v.s. time, where the radius of the large microbubble is 1.6 µm. Simulations with various small microbubble radius ($R_{0}\in$ [0.7,1.1] µm) are conducted, indicated by the error bar, to exclude the influence of the bubble radius on the simulation results. The microbubble coalescence is observed under high flow rate conditions in B-D, and under low flow rate conditions in D.

Fig. 7A presents typical experimental observations acquired at the same location under identical imaging settings. Here, we focus on the influence of the flow rate on the motion of an unequal sized microbubble pair, where the large microbubble barely moves during the ultrasound excitation, and the small microbubble is attracted by the large microbubble. Under high flow rate condition (the large microbubble radius $R_{0}=1.6$ µm, and the small microbubble radius $R_{0}=0.9$ µm), we observe the onset of coalescence during the 50 µs ultrasound excitation; under low flow rate condition (the large microbubble radius $R_{0}=1.6$ µm, and the small microbubble radius $R_{0}=0.95$ µm), we observe that the small microbubble moves in a much slower velocity and eventually cannot reach the large microbubble during the 50 µs ultrasound excitation. The corresponding bubble–bubble distance *d–t* trajectories (Fig. 7B-D, where Fig. 7A is corresponding to Fig. 7C) shows that similar sized microbubble pair under different flow rate could behave totally different, where the microbubble coalescence is observed experimentally under high flow rate condition in Fig. 7B-D, and under low flow rate condition in Fig. 7D when the initial bubble–bubble distance *d* is smaller. The simulation results based on Eq. (3) show that the influence of the microbubble-wall distance h may play a determinable role on lateral bubble motion. For larger h, microbubbles actively migrate toward each other, come into contact and coalesce within 50 µs, even if the initial bubble–bubble distance is relatively large (Fig. 7B, h=1 µm, Fig. C, h=0.8 µm). In contrast, for smaller h (Fig. 7B-C, h=0.1 µm), the enhanced wall-induced drag substantially reduces parallel motion, and the bubbles appear effectively 'frozen' near their initial positions. For bubble pair much closer to each other initially, we always observe coalescence events (Fig. D).

### 3.5 The microbubble motion vertically to the wall

The simulation results indicate that microbubbles get frozen because they are very close the PDMS wall. To elucidate the combined effects of short-pulse ultrasound and flow rate on microbubble vertical motion, it is necessary to consider the forces exerted on the microbubbles by both the acoustic excitation and the laminar flow.

**Fig. 8:**
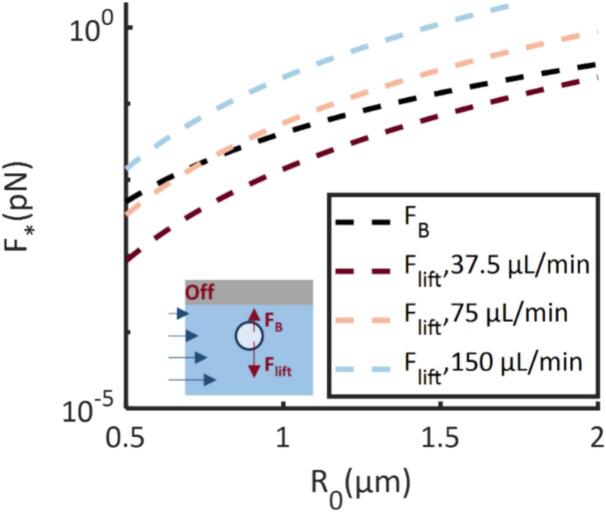
**Forces dominate vertical microbubble motion.** The lift force $F_{\mathrm{lift}}$ v.s. buoyancy force $F_{B}$ over different equilibrium bubble radius.

**Prior to ultrasound exposure**, microbubbles located near the PDMS wall within the rectangular microfluidic channel move predominantly in the x-direction, following the Poiseuille flow profile:

(4) $$u_{x}\left(z\right)=\left(6Qz\right)/\left(H^{2}W\right)$$

with a nearly constant shear rate $\gamma =\left(6Q\right)/\left(H^{2}W\right)$ [71] nearby the wall, where Q is the volumetric flow rate, H and W are the height and width of the microfluidic channel, respectively, and z denotes the distance from the PDMS wall (with z=0 at the top PDMS wall). In the studied scales [72], the measured microbubble velocity reflects the local Poiseuille flow velocity, the microbubbles are transported downstream by laminar flow, and, under these conditions, the Saffman force is negligible, and vertical migration is primarily governed by the combined effects of shear-gradient lift and wall-induced lift forces [73]. A simplified expression for the lift force can be written as $F_{\mathrm{lift}}=0.5\rho \gamma^{2}{\left(2R_{0}\right)}^{4}$, which is counteracted by buoyancy, $F_{B}$ [73]. As illustrated in Fig. 8, at low flow rate (37.5 μl/min), buoyancy dominates the vertical motion (in z-direction), driving microbubbles gradually toward the PDMS wall. With increasing flow rate, the lift force becomes stronger and governs the cross-stream migration, resulting in distal motion from the wall under high flow rate conditions (150 μL/min).

**During ultrasound excitation**, the bubble oscillates and experiences an acoustic radiation force arising from the pressure gradient at the bubble location. In the studied geometry, the bubble experiences radiation forces -V∇P vertically. The pressure gradient ∇P induced by the external ultrasound wave propagating in the positive z-direction leads to primary Bjerknes force $F_{\mathrm{pB}}$ on the bubble, and the pressure emitted by microbubble itself and reflected by the PDMS wall leads to the secondary Bjerknes force $F_{\mathrm{sB}}$ on the bubble. The water–PDMS interface can reflect the acoustic pressure with opposite sign since PDMS is acoustically softer than water, and, thus, promote bubble motion in positive z direction like the primary Bjerkens force [74]. However, when the initial distance between microbubble and the PDMS wall is close, the presence of the PDMS wall with Young’s modulus in Megapascal range can redirect the flow motion during bubble oscillation [[75], [76]] and introduces a hydrodynamic interaction force which can be interpreted as the effect of an image microbubble (hydrodynamic mirror). This interaction leads to periodic, unsteady migration of the microbubble toward the wall and can be described using Kelvin impulse theory [[77], [78], [79]].

Based on this force analysis, the combined effects of short-pulse ultrasound and flow rate on microbubble motion can be understood. Under low flow rate, buoyancy dominates over lift forces, driving microbubbles toward the PDMS wall. As a result, the bubbles become effectively 'frozen' under ultrasound excitation and further increases in pulse length have less influence on microbubble motion in these frozen regions, both parallel and perpendicular to the wall [80].

With increasing flow rate, the lift force can induce reverse motion in the z-direction, driving microbubbles away from the PDMS wall. However, once short-pulse ultrasound is applied, the wall-induced hydrodynamic force can drive the bubbles toward the PDMS wall, thereby reducing the bubble-wall distance, and the corresponding downstream velocity after short-pulse ultrasound. During the first ultrasound burst, microbubbles may therefore remain actively interacting and coalesce into larger bubbles. These larger microbubbles exert stronger secondary Bjerknes forces on one another and can thus remain actively mobile for a longer period. Nevertheless, once a sufficiently thin liquid gap is established, both small microbubbles and isolated large microbubbles become spatially frozen, yet oscillating, bubbles.

## 4 Discussion

This study demonstrates that microbubble cloud dynamics under short-pulse ultrasound in confined flow are affected by the competition between secondary Bjerknes forces and wall-induced drag. Our high-speed visualizations and quantitative analysis reveal two distinct regimes: an actively interacting regime characterized by clustering and coalescence, and a 'frozen' regime in which bubbles exhibit minimal motion under continued acoustic excitation. Both regimes lead to the development of randomly frozen microbubble in sub-wavelength scale, stably oscillating in local region.

The frozen state arises from wall-mediated hydrodynamic drag. During ultrasound excitation, nonlinear bubble oscillations generate a wall-induced interaction force that drives bubbles toward the PDMS boundary. As the bubble–wall gap decreases, lubrication drag increases rapidly and suppresses translational motion parallel to the wall.

Flow rate plays a dual and important role in this process. At low flow rate (37.5 µL/min), buoyancy positions bubbles close to the wall before ultrasound exposure, facilitating rapid establishment of the lubrication-dominated regime. At higher flow rate (150 µL/min), lift forces initially displace bubbles away from the wall, allowing stronger clustering during early bursts. However, repeated short pulses reduce the wall gap and eventually lead to ‘frozen’ bubbles. Pulse length primarily affects the early interaction stage. A longer pulse (100 µs) extends the duration of the attractive secondary Bjerknes force, leading to more pronounced clustering and coalescence within the actively moving region during a single burst. This results in a larger drop in bubble number, consistent with the findings of Zhou et al. (2025) [31], who showed that longer pulse lengths accelerate bubble aggregation. This explains our experimental observations that flow rate and ultrasound pulse duration are coupled parameters for developing frozen microbubbles (Fig. 2D). Under low flow rate (37.5 µL/min), frozen regime can develop even under longer pulse (100 µs). With increasing flow rate (150 µL/min), a shoter ultrasound pulse can drive bubbles actively coalescence. However, when ultrasound is applied, the strong wall-induced hydrodynamic force can drive the bubbles toward the PDMS wall, thereby reducing the bubble-wall gap thickness. Once the lubrication constraint is established and bubbles are 'frozen', ultrasound have a limited influence on bubble translational motion.

Our findings align with and extend those of Zhou et al. (2025) [31] and Memari et al. (2023) [32]. Zhou et al. demonstrated in a flowing phantom that microbubble clustering is the primary cause of temporal non-uniformity in stable cavitation intensity under rapid short-pulse ultrasound. Our study provides a mechanistic explanation by identifying the competition between secondary Bjerknes forces and wall-induced drag as the key physical determinants. We show that clustering is not merely stochastic, but a dynamically controlled process governed by bubble-wall proximity, which is modulated by flow rate and ultrasound.

Complementing these insights, Memari et al. [32] reported that faster microbubble flow (30 ml/min) significantly increases endothelial cell membrane permeabilization compared to slower flow (5 ml/min), attributing this to individual bubbles interacting with more cells. Our results offer a bubble-dynamics-centric perspective, revealing an additional layer of complexity: at high flow rates, bubbles initially exhibit greater mobility and active clustering, potentially concentrating mechanical stress. However, they subsequently become 'frozen' near the wall, a state that may produce sustained, localized shear stress on the endothelium, potentially enhancing long-term outcomes through calcium signaling [[32], [81], [82]].

While our theoretical model captures the key physics governing the transition between bubble states, it is built upon several simplifying assumptions that should be acknowledged. First, the coupling between radial and translational dynamics in our simulation of parallel motion is simplified. Second, the model currently considers only pairwise bubble interactions and does not account for the complex, multi-body effects that arise within dense clusters. Third, thermal effects are neglected.

Nevertheless, despite these simplifications, the model successfully reproduces the experimentally observed transition between actively moving and frozen states and identifies the normalized wall gap as the critical control parameter governing this behavior. It is worth noting that in our experimental geometry, the microbubble radius is two orders of magnitude smaller than the channel height (100 µm). This significant size disparity validates several key assumptions in our force analysis. It confirms that the microbubbles act as point particles relative to the scale of the Poiseuille flow profile, allowing us to accurately describe the background flow velocity using Eq 4. This scale separation justifies the use of standard wall-correction models for the hydrodynamic drag force, derived for a sphere near a plane boundary in an otherwise unconfined quiescent flow or shear flow. The presence of the distant opposite wall (100 µm away) has a negligible effect on the lubrication interaction between a bubble and the nearest (top PDMS) wall.

It is also worth noting that bubble oscillation amplitude is primarily determined by the equilibrium bubble radius R_0_, not by whether the bubble is frozen or actively moving (Fig. 6B). Therefore, frozen bubbles do not oscillate less intensely but remain acoustic active. The key interest of the frozen regime lies not in reduced oscillation, but in the spatial distribution and clustering behavior. Low flow rates promote clustered small frozen bubbles near the wall, whereas high flow rates lead to isolated larger bubbles that eventually also become frozen. This distinction has direct implications for the spatial uniformity of mechanical effects on nearby cells (e.g., sustained local shear stress vs. distributed interactions).

Our findings provide insights into the design of ultrasound-mediated drug delivery and therapy protocols. First, the existence of a flow-rate-dependent 'frozen' state suggests that therapeutic outcomes will be highly sensitive to the local hemodynamic environment. In slow-flowing tumor vasculature [33], [34], microbubbles may rapidly transition to a 'frozen', wall-adherent state. This could be beneficial for achieving sustained, localized mechanical effects on the endothelium, potentially enhancing extravasation of co-administered drugs. Conversely, in higher-flow healthy vessels, bubbles might remain actively mobile and interact with a larger endothelial surface area, as suggested by Memari et al. [32]. This underscores the need for patient-specific or disease-specific ultrasound protocols that account for variations in blood flow. Second, the 'frozen' state may represent a distinct bioeffect mechanism. An immobilized bubble oscillating persistently against the vessel wall could generate sustained, localized shear stress. This might be more effective at triggering specific cellular responses, such as calcium waves or the activation of mechanosensitive ion channels and signaling pathways [[32], [82]], compared to a briefly interacting, free-floating bubble. Future studies should correlate the 'frozen' bubble state with specific biological outcomes to determine whether it is a desirable or undesirable state for different therapeutic applications [52].

In conclusion, the dynamic competition between acoustic radiation forces and near-wall hydrodynamic drag, critically modulated by flow rate and ultrasound pulse sequences, determines the fate of microbubble clouds in confined environments. The transition to a 'frozen' state, governed by the bubble-wall gap, is a key phenomenon to be considered in the rational design of cavitation-based therapies to ensure both efficacy and safety.

## CRediT authorship contribution statement

**Yi Xu:** Writing – original draft, Visualization, Investigation, Formal analysis. **Siyu Luo:** Writing – original draft, Visualization, Investigation, Formal analysis. **Yujie Wang:** Writing – original draft, Visualization, Formal analysis. **Liying Wang:** Writing – original draft, Visualization, Supervision, Formal analysis. **Yuzhe Fan:** Writing – review & editing, Writing – original draft, Visualization, Supervision, Software, Methodology, Formal analysis, Conceptualization. **Fenfang Li:** Writing – review & editing, Writing – original draft, Visualization, Supervision, Methodology, Funding acquisition, Formal analysis, Conceptualization.

## Declaration of competing interest

The authors declare that they have no known competing financial interests or personal relationships that could have appeared to influence the work reported in this paper.

## Appendix A Supplementary data

Supplementary Data 1 Appendix A: Characterization of home-made microbubbles (Fig.S1).

Supplementary video 2 Appendix B: Acoustic field characterization (Fig. S2) and Supplementary Movies S1–S2 for bubble dynamics.
