# Supplementary material for: Sub-wavelength scale randomly frozen microbubble during short-pulsed-ultrasound-driven microbubble cluster dynamics in microfluidic channel
Source: Ultrason Sonochem. 2026 Jun 2;130:107912. doi: 10.1016/j.ultsonch.2026.107912 (PMC13264341; doi:10.1016/j.ultsonch.2026.107912)
Supplement: Supplementary Data 1 — Appendix A: Characterization of home-made microbubbles (Fig.S1). [file mmc1.docx]

**Appendix A**

**Characterization of home-made microbubbles**

**
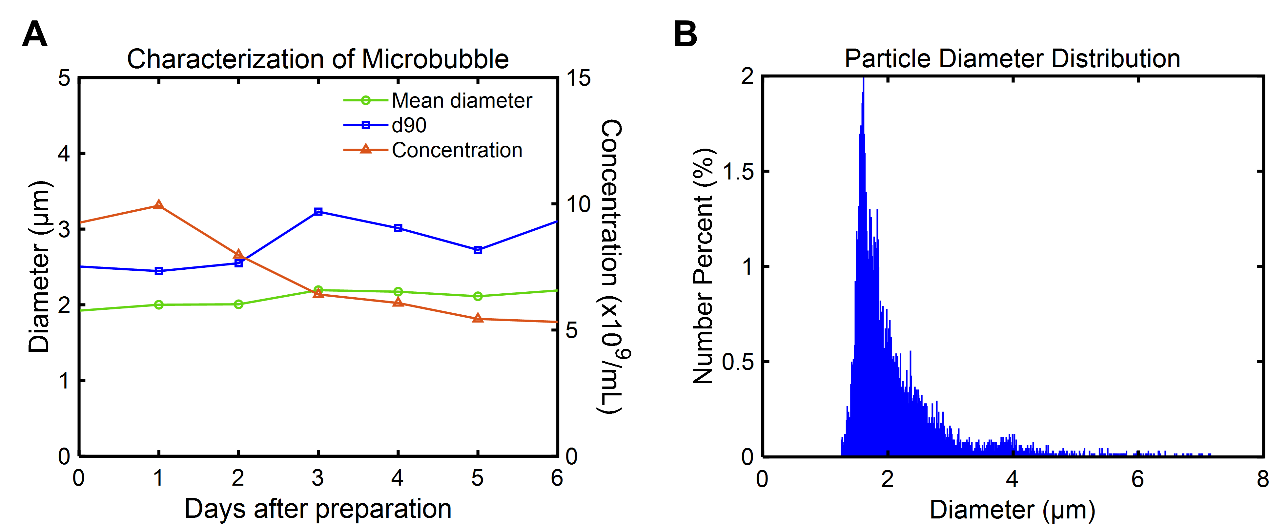
**

**Figure S1. Microbubble Characterization.** (A) Stability of home-made microbubbles with a lipid shell and perfluoropropane core, d90: the size below which 90% of the particles fall within. (B) Size distribution of microbubbles.

**Appendix B: Acoustic field characterization**


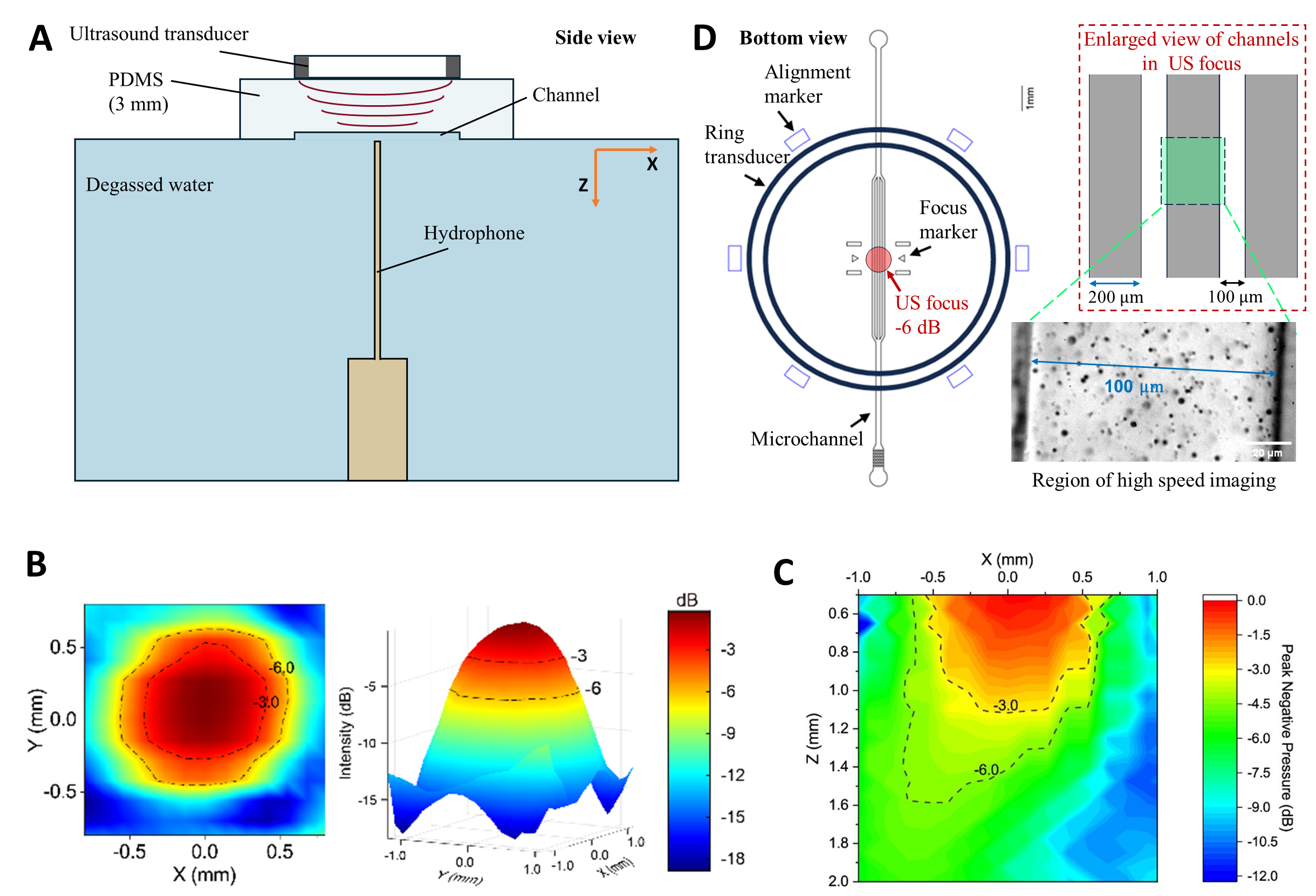


**Figure S2. Acoustic field characterization.** (A) Experimental setup for acoustic field measurement beneath the PDMS top layer. (B) Characterization of the acoustic field produced by the 1.125 MHz ring ultrasound transducer in the X-Y plane after PDMS attenuation. The acoustic pressure is shown in dB relative to the peak value. (C) 2-D contour profiles of the axial pressure fields generated by the ring transducer as measured from hydrophone scans (HNR-0500, Onda Corporation, USA). (D) Bottom view of the geometry for the ring transducer, microchannel, and ultrasound (US) focus. The inset shows the enlarged view of the parallel channels within the US focus. The parallel channels design is for increasing the throughput of cell experiments. The central channel is chosen for high-speed imaging of bubble dynamics throughout the experiment. The channel width (100 µm) and the image frame size is far below the 1 mm-diameter -6 dB focal region of the ultrasound beam and 1.33 mm-wavelength of the ultrasound wave.
